# Supplementary material for: Are carbon emissions trading and green financial instruments synergistic? -Comprehensive quantitative research based on content analysis
Source: PLoS One. 2024 Mar 7;19(3):e0298601. doi: 10.1371/journal.pone.0298601 (PMC10919601; doi:10.1371/journal.pone.0298601)
Supplement: S1 Data — (ZIP) [file pone.0298601.s001.zip › Data/地级市Z_F工作报告.zip!/╔┬╬≈/╙▄┴╓╩╨/└┤╫╘╣1⁄2╓┌║┼í╛┬φ┐╦╩2╛▌═°í┐_2018.docx]

各位代表：

现在，我代表市人民政府向大会报告工作，请予审议，并请市政协委员和其他列席人员提出意见。

一、2017年工作回顾

2017年，是榆林发展史上极不平凡的一年。一年来，全市上下在市委的坚强领导下，深入学习贯彻党的十九大和省十三次党代会精神，紧紧围绕追赶超越和“五个扎实”要求，系统推进稳增长、调结构、促改革、惠民生、防风险各项工作，全力推动一系列补短板、利长远的重大举措、重大工作，全面推开榆林经济社会追赶超越、转型发展的新局面，榆林大地正在发生一场全方位、深层次、根本性的变革。

——经济实力迈上新台阶。实现生产总值3318.39亿元，增长8％；完成全社会固定资产投资1577.1亿元，增长7.5％；完成财政总收入739.57亿元，其中地方财政收入312.97亿元，分别增长51.6％和34.5％，多项经济指标创历史新高，追赶超越和目标责任考核位居全省第一方阵。

——结构调整取得新进展。第三产业增加值增长11.8％，增速全省第一；非能源工业完成产值885.2亿元，增长17.2％；创新驱动发展成果丰硕，高新技术企业产值增长30％，总投资近2000亿元的7大能化标杆项目1加快落地，搭起了转型发展新框架。

——人民生活有了新提升。全市居民人均可支配收入达到22318元，增长9.1％，继续跑赢GDP增速；脱贫攻坚打了一个翻身仗；特别是我们勠力同心、守望相助，战胜了百年不遇的“7·26”特大洪灾，灾后重建取得阶段性成果。

——重大改革实现新突破。“放管服”改革向纵深推进，赵家峁“三变”模式在全省推广，涉煤领域改革、投融资体制改革、园区管理体制改革等关键性改革全面破题，内陆型地区改革创新优势加快释放。

——干部队伍展现新气象。推开事关长远、影响重大的解放思想大讨论、十大战略问题研究2和干部作风大整顿，全市上下思想为之一新、精神为之一振、作风为之一变，心齐气顺、风正劲足的生动局面正在加速形成。

一年来，我们主要做了以下工作：

（一）着力推动经济平稳增长。抢抓宏观经济回暖机遇，加强重点领域运行调度，出台工业促投资稳增长“新19条”、支持民营经济发展“15条”等政策措施，35类重点监测的工业品中24类产量保持正增长，直报工业企业利润增长137.8％，规上工业总产值实现4234.64亿元，增速居全省第一。深入开展项目建设年活动，403个重点项目完成投资714亿元，民间投资增速由负转正。主动适应消费升级新需求，设立1000万元促进商业新业态发展基金，电商销售总额突破10亿元。房地产市场明显回升。实现全社会消费品零售总额472.1亿元，增长11.9％。实施外贸企业培育工程，进出口贸易总额增长28.1％。103个招商引资合同项目总投资突破千亿元，到位资金711亿元。

（二）加快推进产业转型升级。推进传统能化产业清洁化改造，工业技改投资增长141.2％。开展矿业权清理整顿，筹建煤炭转化引导基金。延长靖边填平补齐、神华煤炭综合利用一阶段、陕西精益化工等一批深度转化项目开工建设，佳县天瑞多晶硅、绥德臻梦镁合金、神木泓海荣通制药等一批调结构项目建成投产，战略性新兴产业增加值增长11％。深入实施创新驱动战略，煤基高纯碳研究中心挂牌，粉煤热解工业化取得重大突破。深化农业供给侧结构性改革，山地苹果、大漠蔬菜、中药材等特色产业蓬勃发展，榆林优质农产品标识正式启用，粮食总产量达到165.9万吨，创历史新高。成立市旅发委，举办“陕北榆林过大年”“清爽榆林”等78项特色活动，镇川黑龙潭升格为4A级景区，全年实现旅游综合收入190.8亿元，增长34％。

（三）全面深化重点领域改革。下决心推进“三集中三到位”改革，465项行政许可和公共服务事项进入市民大厦集中办理，“多规合一”在线并联审批平台启用，“12345”便民服务热线一期平台上线，工商登记电子化注册、简化注销、不动产登记改革等工作走在全省前列。全面理顺中心城区规划管理体制，实现“规划一张图、审批一支笔、管理一体化”。扎实推进民生领域改革，城乡居民医保实现“六统一”，市县乡三级诊疗医联体框架初步形成，神木、榆阳、高新区校长职级制改革成效显著。深化财税制度改革，推行市直部门零基预算。加快电力体制改革，完成直供电交易72亿度。

（四）统筹城乡区域协调发展。持续加强中心城市建设，“四馆两中心”3全面启动，1.7万套棚户区改造开工，52个污水直排口彻底治理，顺利通过国家卫生城市复审和省级环保模范城市复查。神木撤县设市。“一县一镇”示范工程加快推进。美丽宜居村庄建设成效显著，榆阳黄家圪公式等5村被评为国家级美丽乡村。盐环定扬黄定边供水提升改建工程开工建设。沿黄公路全线贯通，通建制村公路实现全覆盖，定边县建成“四好农村路”省级示范县。推动振南与脱贫攻坚深度融合，扬榆协作到位帮扶资金6400万元，南六县基础设施和产业发展实现新提升。

（五）持续改善民生福祉。认真落实脱贫攻坚“六个精准”要求，出台40多个行业扶贫政策，投入18.2亿元扶贫资金，实现6.11万人脱贫，162个贫困村出列，定边、横山“摘帽”通过省级核查。横山、府谷、佳县、吴堡、清涧、子洲6县区建成国家义务教育发展基本均衡县。完成中职教育资源整合。全国首家市级“12320”健康咨询服务平台投运，优质医疗资源加快下沉。新增城镇就业2.88万人，转移农村劳动力就业67.7万人。启动民办养老机构医养结合试点，新建农村幸福院150所、城市社区日间照料中心20个。高水平承办省八艺节，《余子俊》等3部剧目获文华优秀剧目奖。

（六）全力守住发展底线。时刻紧绷安全生产这根弦，夯实各级安全生产责任，建立冀晋蒙陕民爆物品安全管理警务协作机制，强化重点领域隐患排查整治力度，煤矿百万吨死亡率历史最低，安全生产形势总体平稳。积极防范化解金融风险，成立榆林金融资产管理公司，推动陕金资和榆能、矿业集团签署债转股协议，银行不良贷款率下降1.92个百分点。启动国家森林城市创建，新增营造林70万亩。实施生态修复工程，治理水土流失面积1153平方公里。红碱淖晋升为国家级自然保护区。以零容忍态度狠抓环境污染治理，榆溪河刘官寨和鱼河断面水质五年来首次由Ⅴ类改善为Ⅳ类，榆林成为全省唯一PM2.5年均浓度值达到国家二级标准的城市。持续强化食品药品安全监管。妥善处置H7N9疫情。不断健全社会治安立体化防控体系，积极化解矛盾纠纷和信访积案，社会大局和谐稳定。

此外，我们在质监、统计、司法、物价、人防、供销、气象、地震、新闻广电、扶残助残、民族宗教、国防军队和政府自身建设等方面都取得了新成绩。

各位代表，在困难多、任务重、挑战大的2017年，我们沉着应对、合力攻坚，取得了来之不易的发展成绩。这是省委、省政府坚强领导、关怀支持的结果，是市委总揽全局、科学决策的结果，是全市广大干部群众同心同德、奋力拼搏的结果。特别是各级干部、各位代表、各位委员，肩负了比以往更沉重的压力，经受了比以往更严峻的考验，付出了比以往更艰辛的努力。在此，我谨代表榆林市人民政府，向全市人民，向各位人大代表、政协委员，向积极参与榆林经济建设的中省驻榆企业和国内外客商，向关心支持榆林发展的社会各界人士，向驻榆部队指战员、武警官兵、政法干警和民兵预备役人员表示衷心的感谢，并致以崇高的敬意！

安不忘危，兴不忘忧。我们清醒地认识到，去年经济增长受能源价格上涨拉动较大，榆林作为一座年轻的资源型城市，人才支撑不足、营商环境不佳、金融生态不优、转型升级步伐不快、生态环保问题较多、安全生产压力较大、城乡区域发展不协调、民生事业发展短板多等老问题依然存在，改革创新能力不强、民间投资活力不足、脱贫攻坚任务艰巨、对外开放水平不高、区域竞争压力加大等新挑战亟待妥善应对。问题是时代的声音，我们将把人民福祉作为奋斗价值，把人民利益作为工作标尺，把人民期待作为行动指针，以“功成不必在我”的胸襟和担当，以“咬定青山不放松”的决心和韧劲，以“抓铁有痕、踏石留印”的作风和行动，一项一项整改，一件一件解决，努力建设一个更加强盛、更加美丽、更加文明的现代化新榆林！

二、新时代政府工作总体要求

党的十九大开启了中国特色社会主义新时代，绘就了决胜全面建成小康社会、夺取新时代中国特色社会主义伟大胜利的宏伟蓝图。榆林正处在大有可为的发展机遇期，我们得“天时”，呼包鄂榆城市群发展规划正式获批，榆林被列为国家现代煤化工示范区，省上专门出台支持榆林高质量发展的意见，榆林在中省发展版图中的地位全方位提升；我们据“地利”，榆林土地广袤，资源富集，产业基础雄厚，区位优势和人文优势突显，发展高端能化、文化旅游、现代物流、特色农业等产业条件优越；我们占“人和”，市委、市政府矢志为榆林人民谋福祉，高瞻远瞩谱新局，事不避难推改革，步步为营抓落实，凝聚了人心、汇聚了力量，全市上下人心思齐、人心思进，正在积聚起榆林追赶超越最强大的力量。

时代潮流、浩浩汤汤。我们要抢抓机遇、乘势而上，在更宽视野、更大格局中统筹谋划榆林未来发展，用习近平新时代中国特色社会主义思想武装头脑，全面落实“三大目标”4和“两个更大”5要求，围绕榆林“三步走”战略部署6，奋力开启榆林新时代加快发展的新征程！重点做好“五个坚持”：

一是坚持创新驱动，着力构建现代化经济体系，加快实现高质量发展。坚定不移走资源转化、产业转型、发展方式转变的路子，按照“优煤、稳油、扩气、增电、强化工、促新兴”的思路，构建能（源）—化（工）—材（料）一体化产业新格局，形成能源化工全产业链竞争优势；坚持创新引领、主动转型，着力突破科技和人才瓶颈，打造现代煤化工、镁铝、新能源、文化旅游、战略性新兴产业、现代服务业六大千亿级产业集群，加快构建与之配套的科技创新、现代金融和人才供给体系，推动“三大变革”，建成世界一流高端能源化工基地。

二是坚持生态优先，着力统筹资源开发与生态建设，加快实现绿色发展。走“保、治、补、用”相结合的路子，推动资源开发与生态建设在时间进度、空间分布、投入产出上协调共进，完善生态补偿机制，落实生态治理责任，实现人与自然和谐发展。践行“绿水青山就是金山银山”理念，大力发展生态经济，建成国家森林城市和国家生态保护示范区，把优良生态作为加速转型的“绿色引擎”，久久为功建设黄土高原生态文明示范区。

三是坚持区域协调，着力形成市域一体化发展新格局，加快实现城乡融合发展。把握人口规律，立足现实条件，打破县区部门利益藩篱，统筹推进新型城镇化和乡村振兴战略，下好全市“一盘棋”。开展中心城市“强基提质三年行动”，实现城市管理大变革、面貌大改善、品质大提升，集聚人流、物流、资金流、信息流，打造陕甘宁蒙晋交界最具影响力城市。以打赢脱贫攻坚战为底线，以农村环境综合整治为抓手，以乡村振兴示范村为载体，以深化农村改革为动力，推动资金和资源进一步向农业农村倾斜，打造全国知名现代特色农业基地和全省乡村振兴示范区。

四是坚持改革开放，着力打造陕西向北向西开放门户，加快实现开放发展。坚定不移向改革聚力，牢牢抓住基础性关键性改革，破除体制机制障碍，打造全国一流营商环境，以改革的新成效开辟榆林发展的新境界。坚持以开放包容的胸怀解放思想、引资引才，深度融入呼包鄂榆城市群和“一带一路”，强力推进引黄、机场、高铁、能源外送通道等事关长远的重大基础设施建设，不断增强国际国内高端资源要素的吸附力和整合力，加快形成全方位开放新格局。

五是坚持发展为民，着力完善基本公共服务体系，加快实现共享发展。坚持把握规律、完善制度、突出重点、市域统筹，深化民生领域改革，推动市域基本公共服务均等化，实现民生发展与经济发展、人口布局、可用财力、人民诉求相匹配。以“群众视角”办好民生实事，着力解决“上学难”“看病难”“养老难”“办证难”“物业乱”等突出问题。以超常举措增加优质民生供给，将教育、医疗、养老等单项民生提高到与大中城市比肩的水平，打造最具影响力城市的新名片。

三、对2018年政府工作的建议

2018年，是贯彻党的十九大精神的开局之年，是改革开放40周年，是榆林能源化工基地建设20周年，更是在市委坚强领导下谋篇布局、打开局面的关键一年。按照市委四届四次全会部署，2018年经济社会发展总体要求是：以习近平新时代中国特色社会主义思想为指导，紧扣追赶超越和“五个扎实”要求，落实“五新”战略任务，聚焦“三大目标”，坚持稳中求进工作总基调，坚持新发展理念，以转型升级为主线，以改革创新为动力，统筹推进稳增长、促改革、调结构、惠民生、防风险各项工作，全力打好防范化解重大风险、精准脱贫、污染防治三大攻坚战，努力推动质量变革、效率变革、动力变革，为陕西追赶超越作更大支撑，为加快陕西经济社会发展作更大贡献。

主要预期目标是：地区生产总值增长7.5％，规上工业增加值增长5％，地方财政收入增长8.3％，全社会固定资产投资增长10％，社会消费品零售总额增长10％，城乡居民收入分别增长7.5％和9％。

围绕上述目标，重点做好七方面工作。

（一）统筹推进促投资稳增长，夯实追赶超越基础。

大力发展实体经济。深入实施降成本行动计划。启动国家电力体制改革试点，开展榆神工业区和长庆靖安油田增量配电业务改革，全市直供电交易突破100亿度，一般工商业电价平均降低10％。全面推开涉企收费清单管理和网上公示制度，开展清理规范中介服务专项行动，积极争取高速公路差异化收费试点，全面实现公路通行费电子发票开具，切实降低企业非税负担。始终将民营经济作为振兴实体经济的关键，不折不扣落实支持民营经济发展的各项政策措施，设立3亿元民营经济发展基金，推行财政补贴民企聘请职业经理人、技术带头人和财务总监制度，继续选聘优秀高校毕业生到民企工作，创造条件支持民企国企协作配套，全年新培育“五上”企业80户。支持国能投、中煤、延长、陕煤、陕投、陕有色、兖矿等企业在榆发展，加快榆能集团、城投集团等市属国企“一企一策”改革，充分发挥国有企业的支撑引领作用。

开展“重大项目攻坚年”活动。投资依然是拉动榆林经济增长的主要动力，完成2000亿元的固投任务是抓好今年经济工作的关键核心。严格落实“五个一”包抓机制，强化挂图作战、排名公示、项目代办等推进制度，用节点倒逼工期、用责任倒逼进度，确保260个重点建设项目6月底前全部开工，大梁湾煤矿、佳县120万吨真空制盐等84个重点项目年内建成。进一步加强前期保障，全力推动陕煤煤炭分质利用、中煤煤炭深加工、延长煤制芳烃、延长榆神煤炭综合利用、未来煤液化一期后续、神华榆林煤炭综合利用二阶段工程6个能化标杆项目开工建设。围绕审批、用地、资金等关键环节，做深做细前期工作，确保完成6000亿元滚动投资审批任务，74个重大前期项目具备开工条件。发挥好政府投资引导带动作用，用PPP模式挖掘社会投资潜力，在交通、电力、市政基础设施等领域谋划、储备、实施一批重大项目。

更大力度招商引智。深入开展“招强引大、招名引优”专项行动，拿出最优质资源和最优惠政策，着力引进一批总部型、旗舰型、建链型项目。强化园区招商主战场作用，积极发展“园中园”“飞地园区”，推行“保安式”跟进和“保姆式”服务，确保60％的县域招商引资项目在园区落地。推行市县两级招商联席会议制度，强化协调服务、跟踪督办、绩效考核机制，全面提升招商引资合同履约率、资金到位率和项目开工率，全年引进中国五百强企业和填补产业空白重大项目不少于30个，实际到位资金超过800亿元。大力实施“榆商回归工程”，吸引更多榆商回乡创业。人才是榆林高质量发展的最大短板，要把招才引智和招商引资放在同等重要位置，打造区域最具吸引力的人才政策体系，营造人尽其才的干事创业环境，让人才引得进、留得住、能出彩。

提高财政金融保障能力。拓展财力来源，加强煤炭等重点领域税收征管，积极争取中省资金、地方政府专项债券和棚改政策性贷款。强化财政资金绩效管理，严控政府一般性支出，集中财力办大事。加快发展现代金融，设立金融服务中心，积极组建榆林金控集团，支持榆林金资争取全国AMC牌照，力争榆林农商银行挂牌，推动汇森煤业、榆能、广济堂、北元化工主板上市，健全市县两级政府融资担保体系，提升金融服务实体经济能力。

强行优化营商环境。深入开展营商环境“十大行动”，推进“多证合一”“证照分离”等商事制度改革，激发市场主体活力，新注册企业增长12％以上。充分运用“多规合一”成果，实现投资项目全流程在线并联审批，确保从项目立项到施工许可60个工作日办结。在榆神、榆横工业区试点推行企业投资项目承诺制。大力支持标准厂房建设，鼓励实施政府配套场平政策，积极推行先租后让、租让结合、弹性年期等土地供应方式，大幅缩短项目实施周期。开展诚信榆林建设专项行动，推行守信联合激励和失信联合惩戒制度，完善企业信用数据库和企业家诚信档案，强力整治“老赖”、非法集资、恶意逃废债、公职人员欠贷等行为，不断优化区域信用环境。以零容忍态度严打黑恶势力，铁腕整治煽动滋事、强买强卖、恶意阻工等违法行为，全力为项目建设、企业经营保驾护航。

各位代表，营商环境差是制约榆林发展的关键症结，已经到了必须做出根本性改变的时刻。我们将刀口向内推改革，以身作则讲诚信，动真碰硬破积弊，以铁的决心、实的举措，迅速改善榆林营商环境！

（二）深化供给侧结构性改革，推动高质量发展。

深入实施创新驱动战略。坚持将创新作为第一动力，积极申报国家创新型城市试点，加快建立企业为主体、市场为导向、产学研深度融合的科技创新体系。深入实施科技型企业“333”行动计划，培育科技型中小企业50家，新认定高新技术企业5家。围绕粉煤干馏工业化、煤热解气化一体化等重点领域，加快推进原始创新和集成创新。启动建设国家级煤炭分质利用、煤基材料等创新中心，新建各类创新平台12个。积极培育氢能、大数据、集成电路、临空经济等新产业、新业态，为转型升级注入新活力。

促进传统产业提质增效。强化环保、安全倒逼，下决心出清一批长期停产的“僵尸企业”，破除无效供给，腾出发展空间。实施新一轮重大技术改造升级工程，加快兰炭干熄焦、PVC无汞催化、金属镁竖罐改造等技术推广应用，做好现役机组升级改造和小火电关停替代工作。支持红枣、黄芪、海红果等特色农产品加工和服装产业做强做大，打造西部农副产品加工基地和全国羊毛防寒服生产基地。推进商贸服务业提档升级，新培育25个新业态项目，推动万达广场年内开业，支持定边创建国家级电子商务进农村示范县。依托能源化工等优势产业，加快发展研发设计、融资租赁、信息技术等生产性服务业。

加快培育发展新动能。创建国家资源富集地区转型创新试验区，完成30％以上的煤炭矿业权清理整顿，实质运作煤炭转化引导基金，运用市场、经济、法制等手段推动资源深度转化。开工建设靖边400兆瓦光气氢牧多能互补、大唐定边50兆瓦风力发电等项目，下功夫解决新能源发电接入和消纳问题，全年新增新能源装机70万千瓦。编制新能源及半导体、镁铝产业发展规划，创建金属镁国家工程技术研究中心和产业联盟，加快推进天瑞多晶硅二期、陕有色50万吨镁铝合金二期、府谷360万吨氧化铝等项目，打造具有全球竞争力的镁铝合金、半导体材料生产加工基地。组建市旅游投资集团，实施石峁华夏文化、统万城匈奴文化国际化工程，推进黄河国家公园、绥德红色革命教育基地、“三十里铺”、清涧“北国风光”等龙头景区建设，加快红碱淖、白云山5A级景区创建，全力打造陕北印象集中体验地和全国知名自驾游目的地，全年接待游客突破4000万人次。开工建设象道国际、神木西站、定边公铁联运等物流项目，支持绥德“互联网＋物流＋政府服务”基地建设，大力发展无车承运物流、集装箱物流，推动门户经济、枢纽经济和流动经济突破发展。

积极扩大对外开放。深化呼包鄂榆城市群、呼包银榆经济区合作，落实扬榆经济合作框架协议，启动乌兰木伦—大柳塔蒙陕合作试验区建设，积极开展能源易货贸易试点，办好“榆林煤博会”、世界“镁都”经济论坛，加强对外经贸合作与人文交流。积极申报一类航空口岸，启动创建B型保税物流中心，简化检验检疫和通关流程，设立海外商务代表处，营造便利进出口环境。持续抓好“外贸企业培育工程”，鼓励企业开展跨境电商、大宗商品期货交易等自营出口业务，积极参加境内外各类交易会、展销会、博览会，加快榆林好产品“走出去”步伐。

各位代表，今年，市委、市政府作出了建设科创新城的重大战略决策。我们将按照“一年定格局、两年打基础、三年出形象”的要求，创新开发模式，深化省市共建，完成各项前期工作，开工一批标志性建筑，启动榆林学院迁建和开放型大学建设，吸引各类创新资源布局榆林，举全市之力打造引领榆林转型升级、提升城市影响力的创新之城、未来之城！

（三）以乡村振兴战略为统领，推进农业农村现代化。

打好精准脱贫攻坚战。下足绣花功夫，强化精准帮扶，全年按现行标准实现11.4万人脱贫，411个贫困村出列，绥德、米脂、吴堡3县“摘帽”。继续加大财政投入力度，市本级投入增幅不低于20％。强化产业、就业和生态扶贫，因地制宜推进光伏扶贫、山地苹果等产业扶贫模式，确保有劳动能力的贫困群众稳定增收。稳妥推进易地移民搬迁和危房改造，完善“五位一体”兜底保障机制。扶持壮大集体经济，健全贫困户与非贫困户统筹机制，实现贫困村与非贫困村协调发展。深化扬榆对口扶贫协作和国家科技部、卫健委定点扶贫工作，深入开展“万企帮万村”行动，完善“三位一体”大扶贫格局。开展脱贫攻坚作风建设年活动，集中解决责任落实不到位、工作措施不精准、资金管理使用不规范等突出问题，杜绝弄虚作假、数字脱贫。强化扶贫不扶懒的制度设计，采用生产奖补、劳务补助、以工代赈等方式，引导贫困人口勤劳致富，消除“精神贫困”，不让党的好政策成为养懒汉的大锅饭。

推动农业提质增效。按照“稳粮、强畜、优菜、扩果、兴药”的思路，推进畜牧业、特色林果业业态升级，支持发展设施农业、功能农业等高效产业，新增山地苹果10万亩，改造红枣低产园10万亩，粮食总产量稳定在145万吨左右。设立农业产业化基金，加快发展农业旅游、文化创意、健康养生等新业态，推动农产品产加销一体化发展，农产品加工业产值比提高到0.55∶1。实施质量兴农战略，建立优质农产品目录，创建2个农产品质量安全监管示范县，打造一批以“榆林”冠名的国际国内知名农产品品牌。推进丘陵山区农林机械化示范创建，开展轮作休耕制度试点，建设高标准基本农田24万亩，推广高效节水示范1.5万亩，不断提升农业生产能力。

促进城乡融合发展。坚持规划先行、示范带动、分类推进，统筹村庄布局、产业发展、基础设施和公共服务，编好市县两级乡村振兴战略规划。启动建设150个乡村振兴示范村，总结推广不同自然本底和发展基础的乡村振兴模式，避免一哄而上、千篇一律。启动实施农村人居环境整治三年行动，以农村垃圾、污水治理和村容村貌提升为主攻方向，加快补齐影响农村居民生活品质的短板。启动农村通村通组沥青（水泥）路、安全饮水巩固提升和自然村通动力电“三提升”工程，全年完成30个村天然气入户，巩固提升20万人饮水安全，“四好农村路”示范总里程突破1000公里。

激发农村发展活力。落实农村土地“三权分置”制度，引导农村土地经营权规范流转。实现县级农村产权交易平台全覆盖。启动实施“三变”改革三年行动计划，今年每个乡镇至少打造1个“三变”改革示范村。实行传统小农户和新型农业经营主体扶持并重政策，培育新型职业农民2000名。鼓励离岗、退休党政干部回乡任职，允许农技人员兼职取酬，扶持培养一批农业职业经理人、经纪人、乡村工匠，吸引支持企业家、专家学者、医生教师等社会各界人士投身乡村振兴事业。传承优秀乡土文化，破除陈规陋习，广泛开展好媳妇、好儿女、好公婆等评选活动，提升文明素质，弘扬时代新风。

各位代表，党的十九大提出实施乡村振兴战略，农村大发展开启了新篇章。市财政将每年安排10亿元，用于乡村振兴，引导带动更多更优质资源流向农业农村，真正让农业更兴旺、农民更体面、农村更美丽！

（四）坚持市域一体，提高新型城镇化水平。

提升中心城市功能品质。高标准完成第五版城市总规修编，组建城市规划管理专家顾问团，强化城市建筑规划要素管控，为建设最具影响力城市谋好篇、布好局。借力省十七运东风，加快补齐中心城区公共服务和基础设施短板。6月底前开工建设“四馆两中心”。规划建设城市绿道和慢行系统，实施红山路拓宽改造等36个市政道路工程7。建设西沙7万吨水厂等16个市政公用项目8，改造升级老城区地下管网。开展加装电梯、屋面修缮等老旧小区改造试点。加快榆溪河生态长廊二期工程9建设进度，建成10个社区公园10，打造十分钟市民健身休闲圈。抢抓国家新一轮三年棚改政策机遇，加快推进榆阳河、肤施路南延、老城内外等棚改工程，采取市场化等多种方式，新谋划实施一批棚改项目。启动榆溪河、沙河、榆阳河综合治理和芹河试验段建设任务，彻底消除城市黑臭水体，让“四河”成为城市新景观。建设20万亩环城绿化生态圈，加快实现中心城市“四季长青、三季有花”。全面提升城市精细化管理水平，抓好“一巩固六创建”工作，完成“一支队伍管执法”改革，下功夫治理停车难、占道经营、露天烧烤等突出问题，下决心解决中心城区产权证办理历史遗留问题。坚持规划引领、保护优先，高起点编制古城开发保护规划，启动古城游客服务中心和东城墙修复工程，做好“文化＋”“旅游＋”文章，擦亮国家历史文化名城的金字招牌。

统筹区域协调发展。启动国土空间规划编制，做好国土三调和林地变更调查，划定生态红线、安全红线、畜禽禁养区，明晰市域空间发展布局。深入推进长城沿线城镇带建设，进一步引导人口、产业集聚。启动沿黄生态城镇经济带建设，安排1亿元专项资金，实施“八大工程”，加快沿黄公路“三改二”和通黄路建设，打造新的区域经济增长带。支持县市区发展首位产业，提升神木、靖边、绥德三个副中心城市辐射带动能力，推动县域经济争先进位。强化园区作为县域经济主战场地位，出台园区改革创新发展实施意见，压缩三分之一以上的园区建制，推开人事薪酬制度改革，激发园区发展活力。支持榆横、榆神、锦界、靖边能化等园区申报省级开发区。继续推动振南与脱贫攻坚、灾后重建深度融合，高标准建设绥德大理河分洪隧洞、子洲大理河段防洪能力提升工程，绥德、米脂、子洲三县供水工程年内通水。深化神木国家新型城镇化试点和大柳塔小城市综合改革试点。精心组织省级文化旅游名镇和市级重点镇建设，集中力量打造一批以高家堡旅游小镇、白云山春风十里小镇为代表的特色小镇。

提高基础设施支撑水平。机场方面，加快榆阳机场改扩建，完成飞行区建设，开工航站区工程；启动神木、靖边通用机场建设。交通方面，积极配合做好青银高铁前期工作，全力推动延榆高铁年内开工；加快蒙华、靖神铁路建设，推动冯红铁路年内开工；抓好绥延高速、G338府谷过境线等国省干线公路建设，确保吴定高速、绥德过境公路年内建成通车，力争佳米高速具备开工条件。水利方面，积极推进黄河引水指标置换，开工建设东线引黄工程，加快盐环定扬黄定边供水工程建设，协调启动黄河粗泥沙集中来源区拦沙工程，统筹推进矿井水有效利用。电力方面，规划建设陕北—关中第三、第四条输电线路，开工建设陕北—武汉输电通道、靖边泛海红墩界电厂，建成陕能赵石畔电厂、榆能横山电厂。信息化方面，成立大数据公司，统筹建设智慧城管、智慧安监、智慧公安、智慧医疗等信息平台，积极推进市民一卡通整合工作；深入实施“光网榆林”工程，4G信号覆盖率达到95％以上，建制村光纤覆盖率达到100％。

（五）持续改善生态环境，加快建设美丽榆林。

加强生态保护与修复。深化国家森林城市创建，推进“林业建设五年大提升行动”，实施城市绿化、绿色长廊、沙区治理提升等重点生态工程，全年完成营造林60万亩，义务植树1000万株。改良、丰富绿化树种，评选确定“市树”“市花”。夯实各级河长（湖长）责任，扎实推进无定河全线综合治理，榆阳、横山、米脂、绥德、子洲、清涧6县区防洪工程完成70％工作量；启动大理河流域生态治理，打造黄土高原生态文明示范区建设样板。新建、加固、维修淤地坝800座，全市治理水土流失1100平方公里。加快推进红碱淖申报第二批流域生态补偿试点，争取设立定边苟池湿地保护区。实施天然林保护和森林生态效益补偿制度，做好封山禁牧、森林防火、林业有害生物防治，切实保障森林生态安全。

打好污染防治攻坚战。开展第二次全国污染源普查，完成环境容量研究，全面摸清污染源“底数”和环境承载力。切实做好中省环保督察问题整改落实和“回头看”工作，完成园区“两场”建设任务，坚决关停环保不达标企业。启动大气污染防治三年行动，开展挥发性有机物、扬尘、机动车尾气污染专项整治，完成30万千瓦以上燃煤机组超低排放改造。全面落实“土十条”，开展土壤污染详查，加强土壤污染管控和修复，提升工业固废处置能力，推进榆阳、定边病死畜禽无害化处理项目。深入实施“水十条”，织密水质监测网络，加大河道整治力度，强化省际界河、界湖环境污染治理。

完善绿色发展长效机制。严格落实主体功能区定位，编制产业准入负面清单，严守生态保护红线。建立矿产资源开发生态补偿机制，夯实企业生态修复责任，有序治理采煤沉陷区、火烧区。出台工业“三废”综合利用及处置管理办法，分类推进排污许可“一证式管理”，顶格执行生态环境损害赔偿制度。支持神木国家生态文明先行示范区建设。组建生态环境治理投资公司，大力发展节能环保、清洁能源、共享经济等环境友好型产业。

（六）补齐民生发展短板，提升人民群众获得感。

办好人民满意教育。深化教育综合改革，推进“管、办、评”分离，推开中小学校长职级制和教师“县管校用”等改革举措，稳步提高教师福利待遇。深入实施新一轮学前教育三年行动计划，出台民办幼儿园管理办法，启动114所公办幼儿园建设，年内普惠性幼儿园占比达到80％以上。推动义务教育均衡发展，支持绥德创建国家义务教育发展基本均衡县，开工中心城区25个校建工程11，基本消除66人以上超大班额。加快发展职业教育，落实30％地方教育费附加用于职业教育政策，支持榆林职业技术学院建设国内一流院校和一流专业。下功夫引进省内外优质教育资源和品牌，大力支持民办教育发展，带动办学条件、质量双提升，努力让榆林的孩子不再为求学远赴他乡，努力让家长不再为抚育子女两地奔波。

推进健康榆林建设。深化公立医院改革，完善现代医院法人治理制度，出台人事薪酬制度改革方案，组建1—2家紧密型医疗联合体。健全乡村医疗服务体系，建成标准化乡镇卫生院40所、规范化村卫生室200个。加强社区卫生服务中心和全科医生队伍建设，打造城市15分钟就医圈。启动建设市中医院、疾控中心、卫生监督所、妇幼医院和感染性疾病诊疗中心，确保市精神卫生中心年内建成投用。引进西安红会医院、西北妇幼院、西安儿童医院等知名医院和重点专科进驻榆林。加强重大疾病防控，提升公共卫生服务水平。深入实施全民健身计划，积极举办黄河漂流、马拉松、沿黄自行车赛等全国性、国际性赛事，组织参加好省十六运，全力筹备省十七运，促进全民健身和全民健康深度融合。

健全社会保障体系。全面加强公共就业和创业服务体系建设，申报创建省级创业型试点城市，突出做好高校毕业生、城镇就业困难人员、农村转移劳动力和退役军人等重点群体就业工作，新建3个市级创业孵化园区，开展就业创业培训1万人次，新增城镇就业2.85万人。实施全民参保计划，扩大中小企业员工、灵活就业人员、农民工参保面，完成城乡居民基本医保制度整合。修订“十三五”养老规划，出台支持社会资本兴办养老机构政策措施，建立农村养老互助幸福院发展长效机制，福彩公益金50％以上投入养老事业，全年新建农村幸福院120个、社区日间照料中心10个。积极发展医养结合试点，启动乡镇养老、卫生机构融合发展试点改革，探索建立养老长期护理保险制度。全面落实拥军优属和优抚政策，不断增进军政、军民团结。健全农村留守儿童、困境儿童、妇女、老年人关爱保障体系，加强残疾人康复服务，让弱势群体充分享受党和政府的温暖。

推动文化繁荣兴盛。深化全国文明城市创建，开展“厚德榆林”“文明家庭”评选等各类活动，力争在第六轮全国文明城市年度测评中进入前30位。启动广播电视塔迁建。完成榆溪楼陈列布展。切实做好文物古迹、非物质文化遗产、红色革命旧址等重要文化载体保护开发。加快推进县区“三馆一院”等公共服务设施建设，提升文化广场、农家书屋等公共文化资源共建共享水平，打造一批“十里公共文化服务圈”。深入挖掘榆林多元文化资源，加快发展出版、影视、动漫、文创等特色文化产业，力争引进2家国内文化百强企业，开工建设2个文化产业园，不断提升榆林文化软实力。

各位代表，人民对美好生活的向往，就是我们的奋斗目标。今年我们将全面推开民生实事公开征集制度，让老百姓关心的事成为政府工作的重点，让人民群众生活得更舒心、更顺心！

（七）坚持底线思维，打好防范化解重大风险攻坚战。

防范化解金融和政府债务风险。坚持用市场化、法制化手段化解不良贷款，夯实各级政府属地责任，用好10亿元债务风险补偿基金和5亿元债务化解基金，支持各类资产管理公司收购处置不良资产，切实加大金融机构不良贷款核销力度，确保银行不良贷款率下降到4％以内。建立金融风险及非法集资监测预警平台，加强类金融机构和新兴金融业态监管，坚决守住不发生系统性金融风险底线。加强政府性融资平台和政府债务规模限额管理，支持县市区依法依规争取政府债务置换债券，有序化解存量债务，坚决堵住违规举债的口子。

守住安全生产底线。严格落实安全生产责任制，强化企业主体责任，积极推进企业安全生产标准化建设，广泛开展安全生产宣传教育培训。深入开展道路交通、煤矿、危险化学品安全“三项攻坚行动”，坚决遏制重特大安全事故发生。深入推进食品药品安全综合整治，持续强化对“三小”行业、集贸市场、学校食堂、农村红白事宴席等重点领域的监管，确保人民群众“舌尖上的安全”。进一步健全应急管理体系，推进防灾减灾救灾体制改革，提高处置突发事件能力。

创新社会治理体系。深入推进“四社联动”，新建30个城市社区，启动200个农村社区建设试点，引领带动全市社区治理法治化、科学化、精细化。进一步强化信访工作，全面夯实各级信访责任，依法打击缠访闹访行为，严惩一批典型非访人员。做好矛盾纠纷排查化解，抓小抓早抓苗头，确保矛盾纠纷不激化、不扩散、不上交。深化“平安榆林”建设，强力开展“扫黑除恶”专项斗争，启动实施“雪亮工程”，持续强化民爆物品管控，加强立体化社会治安防控体系建设，切实保障人民群众生命财产安全，确保社会大局和谐稳定。

四、切实加强政府自身建设

中国特色社会主义进入新时代，对政府工作提出了新要求。我们要坚决维护习近平总书记党中央的核心、全党的核心地位，坚决维护以习近平同志为核心的党中央权威和集中统一领导，在省委、省政府和市委的坚强领导下，扎实推进政府机构改革，加快政府职能转变，不断改进政府工作，努力建设人民满意的服务型政府。

建设公正法治政府。把法治理念贯穿政府工作全过程，进一步完善政府权力清单和责任清单，切实做到“政府权力进清单、清单之外无权力”。科学编制政府立法计划，推动重点领域立法。全面推开“双随机、一公开”制度，严格规范行政执法行为。认真落实“三重一大”决策机制，将专家论证作为重大决策的前置性程序，努力提升政府科学决策水平。依法向人大报告重大事项，建立人大代表、政协委员列席政府常务会议制度。自觉接受人大、政协监督，认真听取各民主党派、工商联和无党派人士意见，切实提高人大代表建议和政协委员提案办理质量。深化政务公开，全方位开展“百姓问政”，用真刀真枪的问政问出干部的新作风、政府的新形象。

建设务实高效政府。启动“狠抓落实年”活动，推行台账管理和重点工作牵头部门向市政府常务会议述职制度，压实市直部门和县市区责任，推动各项工作落地落实。强化督查问责，组建由离岗县级领导任组长的专业督查组，实行定期督查调度和红黄牌制度，坚决整顿顶着不干、拖着不办、有令不行、有禁不止等德不配位、懒政怠政行为。启动“最多跑一次”改革，完成县乡政务服务中心标准化建设，投用政务服务“一张网”，让数据多跑路、群众少跑腿，把百姓对政府的满意度提升到一个全新水平。

建设清正廉洁政府。扎实开展“不忘初心、牢记使命”主题教育活动，严格落实全面从严治党主体责任，让党员干部知敬畏、存戒惧、守底线。认真落实中央八项规定实施细则精神，坚决防范“四风”问题反弹回潮。启用公共资源交易平台，10大类事项全部进入平台公开交易。紧盯工程建设、国资监管、资金分配使用等重点领域，加强审计监督和廉政风险防控。全力支持配合监察委员会工作，坚决纠正各种不正之风，努力营造风清气正的政治生态。

各位代表！榆林是我们共同的家园，把榆林建设得更加美好是我们义不容辞的责任和使命。让我们更加紧密地团结在以习近平同志为核心的党中央周围，高举习近平新时代中国特色社会主义思想伟大旗帜，在市委的坚强领导下，团结依靠380万榆林人民，万众一心、砥砺奋进，不断夺取新时代榆林追赶超越、决胜小康的新胜利！
